# Editorial: Host-Pathogen Interaction in the Central Nervous System

**DOI:** 10.3389/fcimb.2021.790761

**Published:** 2021-12-24

**Authors:** Tatiana Barichello, Federico Iovino

**Affiliations:** ^1^ Department of Psychiatry and Behavioral Sciences, University of Texas Health Science Center at Houston, TX, United States; ^2^ Graduate Program in Health Sciences, University of Southern Santa Catarina (UNESC), Santa Catarina, Brazil; ^3^ Department of Neuroscience, Karolinska Institutet, Stockholm, Sweden

**Keywords:** Host-pathogen interaction, Central Nervous System, bacteria, viruses, parasites

Meningitis is an inflammation of the meninges that cover and protect the brain and the spinal cord. This inflammation occurs upon an infection of the Central Nervous System (CNS), and etiological causes of infection can be bacteria, viruses, and in rare cases parasites. Pathogens in the brain encounter different types of cells, such as microglia, the resident macrophages of the brain, neurons, the fundamental cellular units responsible for electrical and chemical signaling, astrocytes, cells in close contact with the blood-brain barrier that participate in immune responses, and other infiltrating immune cells. The scope of this Research Topic was to shed light on all various types of interaction that the different etiological agents of meningitis (bacteria, viruses, parasites) take with the different cell types of the brain during the pathogenesis process.

## Parasitic Meningitis

Meningitis caused by parasites does not always obtain enough attention from the scientific community. Parasites like *Toxoplasma gondii* can be resilient and have the capacity to cause lifelong chronic infections requiring continuous immune responses by the host. Bergersen et al. have provided a comprehensive study of how targeted transcriptomic analysis using mouse models of chronic *T. gondii* infection can reveal differences in the host and parasite gene expression, giving tremendous insights into how further understanding the neuropathology aspects of chronic *T. gondii*-CNS infection (Bergersen et al., 2021).

*Angiostrongylus cantonensis* is another parasite that can cause meningitis, in particular the so-called eosinophilic meningitis. Yet, the pathogenesis of meningitis caused by *A. cantonensis* remains poorly understood. Zhang et al. have shown that upregulation of C-X3-C Motif Chemokine Ligand 1 (CX3CL1) in the brain tissue leads to the recruitment of natural killer (NK) cells into the CNS which of course should help the host in fighting the infection; however, this infiltration into the brain causes a worsening of the host CNS conditions (Zhang et al., 2021). Interestingly, Zhang et al. have observed that by neutralizing CX3CL1 and depleting NK cells, brain injury was alleviated, therefore proposing a new therapeutic intervention to protect the CNS against *A. cantonensis* infections (Zhang et al., 2021).

## Viral Meningitis

Meningitis can also be caused by certain viruses. Dengue virus (DENV) is transmitted by Aedes mosquitoes to humans and is a serious threat worldwide. The main clinical problem is that, up to today, there is not an effective drug against dengue infections. Shen et al. have observed that metoclopramide (MCP), an antagonist of the dopamine 2 receptor (D2R) promotes an impairment of DENV-double-stranded RNA replication and provided promising results regarding the use of MCP as therapy to reduce DENV-induced neuronal damage (Shen et al., 2021).

The Japanese encephalitis virus (JEV) can cause meningitis with permanent neurological sequelae and yet, the mechanism employed by JEV for brain invasion from the systemic circulation remained unknown. Zou et al. finally elucidated the mechanism of entering the brain by blood-borne JAV (Zou et al., 2021). The virus uses monocytes to spread in the brain tissue and expand the infection in the CNS (Zou et al., 2021). Furthermore, the extracellular High mobility group box protein 1 (HMGB1) facilitates the immune cell migration across the vascular endothelium of the BBB, which further accelerates the onset of JEV-induced meningitis (Zou et al., 2021).

Viral meningitis represents a significant burden in tropical countries of South America, and Arboviruses, such as Chikungunya, Mayaro, Oropouche, and Zika viruses, are major etiological agents of this disease. Arbovirus-caused meningitis is also frequently associated with severe neurological outcomes. The comprehensive genome-wide transcriptome analysis of human primary astrocytes infected with Chikungunya, Mayaro, Oropouche, and Zika viruses described by Viana Geddes et al, has revealed a common pattern in downregulation of the host innate immune response, antiviral response, and expression levels of inflammatory cytokines associated with interferon stimulation for all the arboviruses tested (Viana Geddes et al., 2021). These findings point towards a co-evolution that all these arboviruses have engaged in developing mechanisms to escape the antiviral response induced by interferon (IFN) (Viana Geddes et al., 2021). Altogether, this expands the knowledge on how the antiviral-IFN pathway can be experimentally modified in order to be effective against Arbovirus-caused meningitis, opening avenues for novel clinical approaches (Viana Geddes et al., 2021).

## Bacterial Meningitis

*Streptococcus pneumoniae* (the pneumococcus) is the leading etiological cause of bacterial meningitis globally (Iovino et al., 2016). Despite access to antibiotics and the introduction of pneumococcal conjugate vaccine programs, mortality from pneumococcal meningitis exceeds 50% in sub-Saharan African countries with high HIV prevalence, and the causes of such high mortality are poorly understood. Wall et al. have interestingly reported that excessive *S. pneumoniae* elongation factor thermal unstable (EF-Tu) protein in the cerebrospinal fluid (CSF) was frequently associated with impaired survival in meningitis in a high HIV prevalence population (Wall et al., 2020). Moreover, EF-Tu can inhibit neutrophil-mediated killing of *S. pneumoniae* in the CSF (Wall et al., 2020). The findings by Wall et al. provide novel important knowledge on how pneumococci avoid essential host innate responses during meningitis pathogenesis (Wall et al., 2020).

Microglia, the resident macrophages of the brain, initiate and drive the inflammatory process during pneumococcal meningitis pathogenesis. Pan et al. have reported that JWH-133, an agonist of G-protein cannabinoid receptor type 2 (CB2) impairs microglial activation and downregulates pro-inflammatory signaling (Pan et al., 2020). Therefore, this important finding suggests that inhibition of microglial activation using CB2 agonists may represent a novel therapy for neuroinflammation modulation (Pan et al., 2020).

Another major etiological agent of bacterial meningitis is *Listeria monocytogenes*. *L. monocytogenes* meningoencephalitis has a mortality rate of up to 50%, and severe neurofunctional sequelae are prevalent. Zbinden et al. have identified that *L. monocytogenes* expressing multiple sequence recognition (hsdS) A causes less damage than when other hsdS genes (B, C or D) are present (Zbinden et al., 2020). On the other hand, the expression of hsdSC and D worsened the disease onset in *L. monocytogenes* meningitis (Zbinden et al., 2020). This observation shows important phenotypical switching that has crucial role in regulating the virulence of CNS infections by *L. monocytogenes* (Zbinden et al., 2020). Nevertheless, further studies are necessary to investigate how this therapy can also be effective in reducing brain injury.

*Streptococcus suis* (*S. suis*) is an important opportunistic pathogen, which can cause septicemia and meningitis in pigs, but also in humans. In the study by Lauer et al, using Gene Set Enrichment Analysis (GSEA), 18, 28, and 21 enriched hallmark gene sets (GSs) were identified for infected human choroid plexus (CP) epithelial papilloma (HIBCPP) cells, primary porcine CP epithelial cells (PCPEC), and in the CP of pigs affected by *S. suis* ST2 meningitis, respectively of which 8 GSs overlapped among the three different sample sets (Lauer et al., 2021). Most of these GSs were reported to be involved in cellular signaling, host immune and inflammatory response (Lauer et al., 2021). This finding clearly suggests that *S. suis*-infected human and porcine CP epithelial cells share similar cellular processes in the context of host inflammatory response (Lauer et al., 2021).

**Figure 1:**
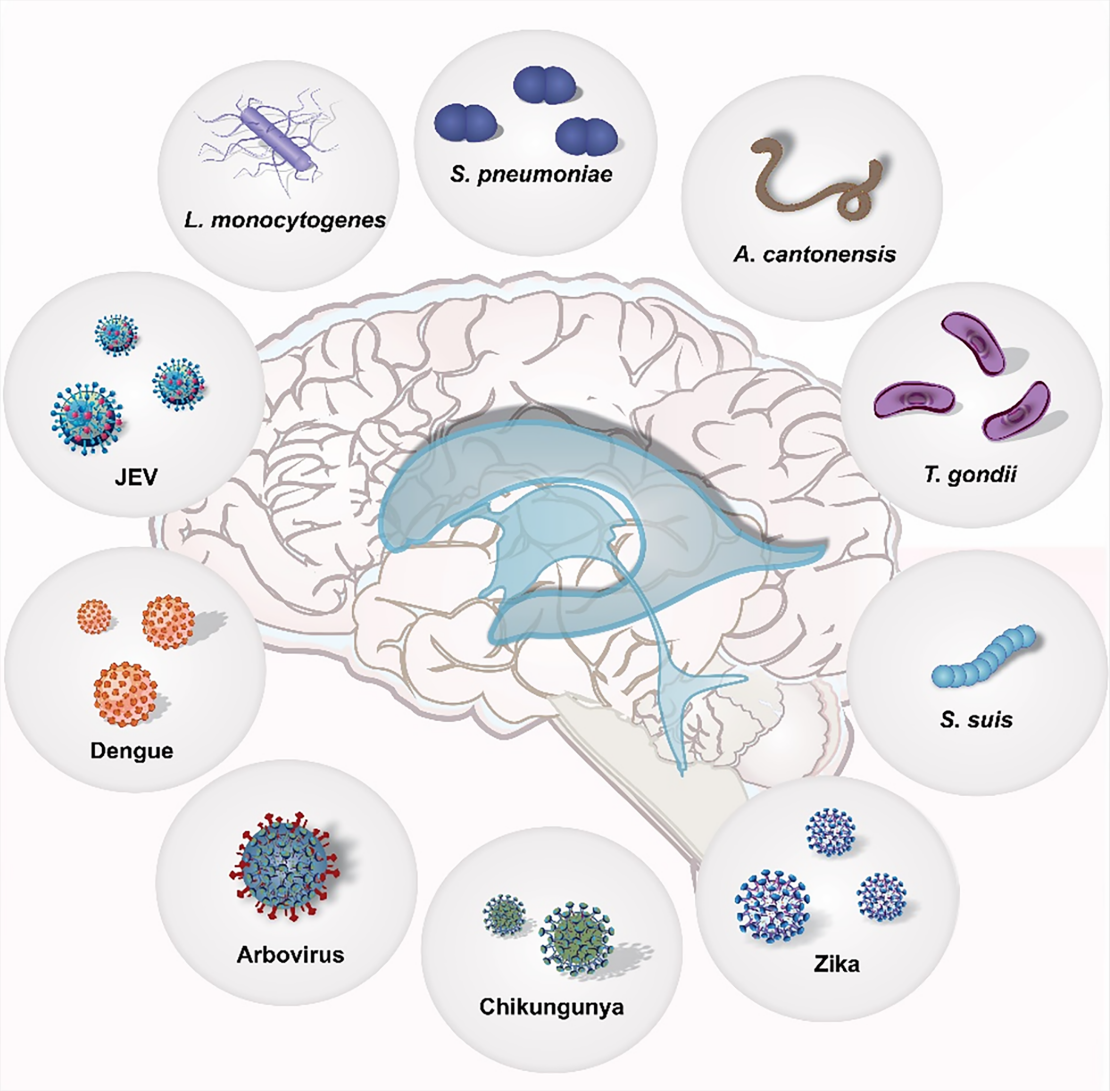
The host-pathogen interaction in the Central Nervous System.

The pathogens covered in the study subject “Host-pathogen interaction in the Central Nervous System” are summarized in **Figure 1**.

## Concluding Remarks

The Research Topic “Host-pathogen interaction in the Central Nervous System” also contains important review articles focused on i) the trans-endothelial trafficking of *S. pneumoniae* across the BBB endothelium (Anil and Banerjee, 2020), ii) the molecular details of a broad array of CNS responses to the bacterial cell envelope and novel approaches to improve clinical outcome (Maccain and Tuomanen, 2020), iii) the benefits and harms of the use of adjunctive steroid therapy to reduce neuroinflammation in meningitis patients (Gundamraj and Hasbun, 2020), iv) the beneficial or detrimental role of DNA sensors in viral CNS infections (Jeffries and Marriott, 2020).

## Author Contributions

TB and FI equally contributed to this editorial article. All authors contributed to the article and approved the submitted version.

## Funding

This work was supported by The University of Texas Health Science Center at Houston. TB has received a grant from the Alzheimer’s Association (AARGDNTF-19-619645) and the National Institutes of Health/National Institute on Aging (NIH/NIA grant 1RF1AG072491). FI is funded by Karolinska Institutet Faculty Board, the Swedish Research Council (grant nr. 2020-0261), Bjarne Ahlström Foundation for Research in Clinical Neurology, Magnus Bergvall Foundation, Tore Nilson Foundation, Clas Groschinsky Foundation, and HKH Crown Princess Lovisa’s Association for Pediatric Research.

## Conflict of Interest

The authors declare that the research was conducted in the absence of any commercial or financial relationships that could be construed as a potential conflict of interest.

## Publisher’s Note

All claims expressed in this article are solely those of the authors and do not necessarily represent those of their affiliated organizations, or those of the publisher, the editors and the reviewers. Any product that may be evaluated in this article, or claim that may be made by its manufacturer, is not guaranteed or endorsed by the publisher.
